# Measurement practices in hallucinations research

**DOI:** 10.1080/13546805.2021.1999224

**Published:** 2021-11-08

**Authors:** David Smailes, Ben Alderson-Day, Cassie Hazell, Abigail Wright, Peter Moseley

**Affiliations:** a Department of Psychology, Northumbria University, Newcastle upon Tyne, UK; b Department of Psychology, Science Laboratories, Durham University, Durham, UK; c School of Social Sciences, University of Westminster, London, UK; d Center of Excellence for Psychosocial and Systemic Research, Department of Psychiatry, Massachusetts General Hospital, Boston, MA, USA; e Harvard Medical School, Boston, MA, USA

**Keywords:** Hallucinations, psychosis, measurement, open science

## Abstract

**Introduction:** In several sub-fields of psychology, there has been a renewed focus on measurement practices. As far as we are aware, this has been absent in hallucinations research. Thus, we investigated (a) cross-study variation in how hallucinatory experiences are measured and (b) the reliability of measurements obtained using two tasks that are widely employed in hallucinations research.

**Method:** In Study 1, we investigated to what extent there was variation in how the Launay-Slade Hallucination Scale (LSHS) has been used across 100 studies. In Study 2, we investigated the reliability of the measurements obtained through source monitoring and signal detection tasks, using data from four recent publications. Materials/data are available at doi: 10.17605/osf.io/d3gnk/.

**Results:** In Study 1, we found substantial variation in how hallucinatory experiences were assessed using the LSHS and that descriptions of the LSHS were often incomplete in important ways. In Study 2, we reported a range of reliability estimates for the measurements obtained using source monitoring and signal discrimination tasks. Some measurements obtained using source monitoring tasks had unacceptably low levels of reliability.

**Conclusions:** Our findings suggest that suboptimal measurement practices are common in hallucinations research and we suggest steps researchers could take to improve measurement practices.

## Introduction

Over the past decade, psychology (like many other disciplines, such as oncology; Begley & Ellis, 2012) has faced a so-called ‘replication crisis’, where many findings have been shown to not be replicable (see Nosek et al., 2015). This has been followed by attempts to address these problems by engaging in methodological practices that increase the transparency, reproducibility, and replicability of psychological science (Nelson et al., 2018).

Initially, attempts to address the causes of the ‘replication crisis’ focussed on issues such as low statistical power (e.g. Bakker et al., 2012) and flexibility in how data are analysed (e.g. Simmons et al., 2011). More recently, it has been argued that the measurement practices psychology researchers engage in warrant increased attention, as accurately measuring our constructs-of-interest is a foundational part of psychological science (de Beurs & Fried, 2021). That is, there is little value in running a high-powered study, in which data are analysed in a transparent, reproducible manner, if we have not effectively measured the variables we are interested in (Flake & Fried, 2020). The difficulties inherent in measuring hallucinations and hallucinatory experiences – as a result, for example, of the complexity and range of experiences participants report (Woods et al., 2015) – mean that it should be especially valuable for there to be a focus on optimal measurement practices in hallucinations research.

Good measurement practices are typically adopted during the initial development of self-report measures of psychological variables, with researchers providing evidence that these measures are reliable and valid assessments of the construct-of-interest. However, it appears that this good practice often does not extend beyond the initial development of self-report questionnaires (Flake & Fried, 2020). One concern is the “jingle fallacy” (Block, 1995), which refers to situations where scales purporting to measure the same construct actually measure different constructs (e.g. the symptoms measured by different depression scales vary so much that these questionnaires may be measuring different constructs; Fried, 2017). This becomes a problem when trying to synthesise inconsistent findings across apparently similar studies, as it is difficult to discern whether cross-study inconsistency is a result of these studies measuring different psychological constructs, or is a result of other factors (e.g. sampling error).

A second concern is the practice of making modifications to a scale following its publication (e.g. changing a scale’s response options, adding/dropping items, revising scoring procedures; Flake et al., 2017). While there may be good reasons for these modifications (e.g. revising items so they are easier to comprehend), these changes may reduce validity and may allow for a degree of analytic flexibility that using an unmodified scale would have prevented (Flake & Fried, 2020). A final concern is sub-optimal reporting of which measures have been employed and how (e.g. failing to report which version of a questionnaire has been employed). There is evidence that this sub-optimal reporting is quite common (Flake et al., 2017); this is important as it may hide cross-study inconsistency in the scales employed, making it more difficult for readers to be confident that constructs have been measured in a reliable and valid manner.

In contrast to the development of self-report measures, where there is at least an initial focus on good measurement practices, little attention appears to be paid to optimal measurement practices when researchers develop tasks to assess putative cognitive biases. For example, Parsons et al. (2019) have argued that studies that employ novel, unstandardised tasks to assess cognitive biases in clincal samples almost always fail to examine and report the psychometric properties of these tasks. As a result, we often have no knowledge of the reliability of the measurements obtained from these tasks. This is problematic because without knowing how reliably a construct has been measured in a study, readers cannot know how confident they should be in that study’s findings. More problematically, it is possible that this lack of transparency “hides” a situation where the reliability of most of the measurements obtained from tasks that aim to assess cognitive biases is low. This is a concern because, as shown by Parsons et al. (2019), if the reliability of the measurements we obtain from the tasks we employ is low, the level of statistical power achieved in our studies is substantially reduced. This reduction in power increases the likelihood of “false negative” findings (incorrectly accepting the null hypothesis) and (counter-intuitively) the likelihood of “false positive” findings (incorrectly rejecting the null hypothesis; Bakker et al., 2012). Thus, suboptimal measurement practices around our development and use of tasks can directly contribute to the generation of research findings that fail to replicate.

These problematic or questionable measurement practices have been investigated in sexual behaviour (Kohut et al., 2020), addiction (King et al., 2020), and anxiety research (Waechter & Stolz, 2015), but have not yet been examined in hallucinations research. Attending to measurement practices in hallucinations research is likely to be worthwhile, given (a) the nature of the experiences hallucinations researchers are interested in (i.e. they are often difficult for participants to describe and sometimes have overlapping phenomenology with other types of unusual cognitions, such as intrusive thoughts; Morrison, 2001; Woods et al., 2015), which makes these experiences difficult to measure effectively and (b) the frequent use of tasks to measure cognitive biases that may be related to the presence/frequency of hallucinations. Thus, across two studies, this paper aims to examine measurement practices in hallucinations research.

## Study 1 introduction

A common approach in hallucinations research is to examine factors associated with non-clinical hallucinatory experiences (HEs; Larøi, 2012). Adopting this approach allows researchers to try to understand the factors involved in the development of hallucinations, without the confounding effects that medication use and/or the long-term presence of hallucinations may have when investigating hallucinations in participants with mental health problems (Badcock & Hugdahl, 2012).

Many measures of HEs have been developed, but perhaps the most widely used is the Launay-Slade Hallucination Scale (LSHS; Launay & Slade, 1981). Since its initial development, English-language revisions to this 12-item questionnaire have been published at least four times, by Bentall and Slade (1985), Morrison et al. (2000), Morrison et al. (2002), and McCarthy-Jones and Fernyhough (2011). As can be seen in Table 1, over the course of four revisions, the LSHS has changed in important ways, including the wording of items, the response options presented, and the number of items presented. Some versions (McCarthy-Jones & Fernyhough, 2011) have tried to maintain a narrow focus on HEs, while others (Morrison et al., 2000, 2002) have assessed a broader set of unusual experiences, (e.g. vivid daydreaming). Thus, it is possible that these scales measure different constructs. As a result, when using different versions of the LSHS, hallucinations researchers may commit the jingle fallacy, and this may make it difficult for the hallucinations research community to develop cumulative science. That said, each version of the LSHS appears to have at least reasonable levels of construct validity (e.g. in terms of its correlations with related variables).

**Table 1.** Summary of the key features of five versions of the Launay-Slade Hallucination Scale.

Version of LSHS	Key features/revisions
Launay and Slade (1981)	- 12-item scale. - Unidimensional. - Yes/No response options. - Items described as assessing ‘vivid thoughts’, ‘intrusive thoughts’, ‘vivid daydreams’, ‘auditory hallucinations’ and ‘visual hallucinations’.
Bentall and Slade (1985)	- 12-item scale. - Minor revisions to wording of some items. - Response options ranging from ‘Certainly does not apply’ (1) to ‘Certainly applies’ (5).
Morrison et al. (2000)	- 13-item scale. - Minor revisions to wording of some items. - Three items deleted. - Four items introduced. - Response options ranging from ‘Never’ (1) to ‘Almost always’ (4). - Two factor structure: one factor assessing “auditory or verbal hallucinations/daydreaming” and one factor assessing “visual hallucinations/disturbances”.
Morrison et al. (2002)	- 24-item scale. - Two items deleted. - 13 items introduced. - Response options ranging from ‘Never’ (1) to ‘Almost always’ (4). - Three factor structure: one factor assessing “vividness of imagination and daydreaming, one factor assessing “visual disturbances and hallucinations”, and one factor assessing “auditory hallucinations”.
McCarthy-Jones and Fernyhough (2011)	- Nine-item scale. - 15 items deleted. - Response options ranging from ‘Never’ (1) to ‘Almost always’ (4). - Two factor structure: one factor assessing “predisposition to auditory hallucinations” and one factor assessing “visual hallucinations and disturbances”.

The first aim of Study 1, then, was to examine how often different versions of the LSHS have been used in 100 recent publications. The second aim of the study was to investigate how these different versions of the LSHS have been used from study-to-study, in terms of the use of subscale scores, rather than full-scale scores. In addition, we aimed to examine how often, and in what ways, these scales have been modified by researchers, and where modifications were made, whether/how these modifications were justified. Finally, we aimed to record how often too little information was provided in a publication for us to fully understand what scale had been used.

## Study 1 materials and method

### Literature search

We used five articles (Bentall & Slade, 1985; Launay & Slade, 1981; McCarthy-Jones & Fernyhough, 2011; Morrison et al., 2000; Morrison et al., 2002) that described the development of different versions of the LSHS as “seed papers” for our literature search. We used the electronic database Scopus to identify studies that had cited at least one of the five seed papers as of 25/SEP/2020 and then merged these five citation lists. The list was ordered chronologically and, beginning with the most recent publication, the abstract or full text of each publication was reviewed (the full-text of a publication was always read, unless it was clear from the abstract that the publication was a review) to examine whether the study had employed a version of the LSHS, until we had identified 100 studies that had employed an English language version of the LSHS. We excluded from this review studies that employed a translated version of the LSHS, as we did not have the expertise to verify how items have been translated (which was relevant to our coding of whether any items had been revised) studies that reported no other analysis than a factor analysis (as we were unable to code these studies in terms of their use, for example, of subscale scores), and studies that reported no inferential tests.

### Coding of studies

After refining a draft coding system, all studies were reviewed and coded by one author (DS), with 20 studies also being coded by a second author (AW), to establish inter-rater reliability. Acceptable levels of inter-rater reliability were achieved (see osf.io/d3gnk/). We coded each study that had employed an English-language version of the LSHS in terms of the 12 variables outlined in Table 2. While several of the variables should have been predictable based on the version of the LSHS that was cited in a study, we anticipated that the way in which the LSHS was used in a study may not always be consistent with the cited version. The coding system is presented in Table 2.

**Table 2.** Coding system for assessing variation in the use of the launay-slade hallucination scale (LSHS) across 100 studies.

Code	Notes
Which version of the LSHS was cited?	Initially, where more than one version of the LSHS was cited, we intended to establish which version of the LSHS the scale employed most closely resembled. However, many reviewed studies provided little information about the scale they employed, causing us to adapt our coding system. Instead, where more than one version of the LSHS was cited, we recorded the most recent version cited as the scale that was employed, as we assumed that this was the most likely version used.
How many items does the scale consist of?	Where this was not clearly reported, we coded this as ‘unclear’.
How many items were revised?	Where this was not reported, we assumed that no items were revised.
What do the scale response options refer to?	We coded this in terms of level of agreement, how much an item applies to the participant, or frequency. Where this was not clearly reported, we coded this as ‘unclear’.
What does the ‘lowest’ response option refer to?	We coded this variable, as we thought that even when response options may have assessed the same concept (e.g., frequency), they may have employed different response options (e.g., Never versus Very Rarely). Where this was not clearly reported, we coded this as ‘unclear’.
What does the ‘highest’ response option refer to?	We coded this variable, as we thought that even when response options may have assessed the same concept (e.g., frequency), they may have employed different response options (e.g., Almost Always versus Every Day). Where this was not clearly reported, we coded this as ‘unclear’.
What was the ‘lowest’ response option score?	We coded this variable, as we thought that even when response options may have assessed the same concept (e.g., frequency), they may have employed different response options (e.g., Never to Almost Always assessed on a 1-4 Likert scale, or Never to Almost Always on a 0-7 Likert Scale). Where this was not clearly reported/easy to calculate from a table of min-max scores, we coded this as ‘unclear’.
What was the ’highest’ response option score?	We coded this variable, as we thought that even when response options may have assessed the same concept (e.g., frequency), they may have employed different response options (e.g., Never to Almost Always assessed on a 1-4 Likert scale, or Never to Almost Always on a 0-7 Likert Scale). Where this was not clearly reported/easy to calculate from a table of min-max scores, we coded this as ‘unclear’.
How many LSHS scores are used as variables in inferential statistical analyses?	We coded this variable to examine whether, for example, correlations were reported for a full-scale score, as well as two subscale scores. Item-by-item prevalence estimates were not included.
Was a full-scale score used?	We coded this variable in terms of yes/no. A ‘yes’ code was used if a score based on a 12, 13, 24, or nine-item full-length LSHS was used. When coding this variable, we included analyses reported in supplementary analyses, as well as analyses reported in the full-text of the paper.
How many subscale scores were used?	We coded the number of LSHS scores used that were calculated by summing responses to a subset of items from a full-length LSHS (e.g., the five-item auditory subscale of McCarthy-Jones and Fernyhough’s [2011] version of the LSHS). When coding this variable, we included analyses reported in supplementary analyses, as well as analyses reported in the full-text of the paper.
Where revisions have been made to the original scale, is a justification provided for the revision?	We coded this variable in terms of, not applicable, no, partial justification, or yes.

## Study 1 results and discussion

Our literature search identified 694 results/studies. To reach our target of 100 studies, we screened the full-texts of 397 studies, with the oldest published in 2010. Most of these studies employed a correlational design and had recruited a non-clinical sample. However. around 25% recruited a clinical, help-seeking, or voice-hearing sample. In many instances, studies did not report a measure of the internal reliability of the version of the LSHS they employed. However, when they did so, internal reliability was almost always acceptable. The full list of search results, reviewed studies, as well as how they were coded is available at osf.io/d3gnk/, with our findings summarised in Table 3.

**Table 3.** Summary of variation in use of the launay-slade hallucination scale.

Characteristic	Percentage of studies
*Which version of the LSHS was cited?*	
Launay and Slade (1981)	24%
Bentall and Slade (1985)	40%
Morrison et al. (2000)	5%
Morrison et al. (2002)	17%
McCarthy-Jones and Fernyhough (2011)	14%
*How many items does the scale consist of?*	
1	1%
2	3%
3	1%
5	8%
6	1%
9	6%
11	1%
12	44%
13	1%
15	1%
16	3%
20	4%
21	1%
24	4%
Unclear	21%
*What do the scale response options refer to?^a^*	
Yes/No	2%
To what extent the item applies	26%
To what extent you agree with the item	3%
How frequently you have the experience	22%
Unclear	48%
*What were the ‘lowest’ and ‘highest’ response option scores?*^a^	
0-1	3%
0-4	29%
0-6	1%
1-4	20%
1-5	7%
Unclear	41%
*How many LSHS scores are used as variables in inferential statistical analyses?*	
0	2%
1	90%
2	5%
3	1%
5	1%
6	1%
*Was a full-scale score used?*	
No	20%
Yes	80%
*How many subscale scores were used?*	
0	78%
1	15%
2	5%
4	1%
5	1%
*Where revisions have been made to the original scale, is a justification provided for the revision?*	
NA	86%
No	8%
Partial	3%
Yes	3%

^a^ Note that the sum of the percentages here exceeds 100 because one study employed response options that referred to frequency and to how much an item applied.

Several findings are apparent from the 100 studies we reviewed. First, four (Bentall & Slade, 1985; Launay & Slade, 1981; McCarthy-Jones & Fernyhough, 2011; Morrison et al., 2002) of the five versions of the LSHS we used as “seeds” were used quite frequently (24%, 40%, 17%, and 14%, respectively). That is, the field does not appear to have “settled” on a preferred version of the LSHS. The use of multiple versions of the LSHS is important as different versions of this scale appear to measure slightly different constructs. For example, while the Launay and Slade/Bentall and Slade versions include items concerning intrusive thoughts and vivid daydreaming, the McCarthy-Jones and Fernyhough version does not. Second, over and above the use of multiple versions of the LSHS, there was substantial variation in the lengths of the scales employed (14 different item-lengths were reported), ranging from one-item versions to 24-item versions. Third, in many studies, too little information was provided for us to code all of the variables we intended (e.g. response options were reported in only 52% of studies). It could be argued that it is unnecessary for authors to provide this information, as long as they cite the version of the LSHS that they have used. However, in some of the studies we reviewed, it appeared that while one version of the LSHS was cited, the response options employed were those used for a different version of the LSHS. For example, in nine of the 11 instances where authors reported using the Launay and Slade (1981) version of the LSHS and reported the response options presented to participants, the response options were not those used in the 1981 version of the LSHS. Meanwhile, in two of the 22 instances where authors reported using the Bentall and Slade (1985) version of the LSHS and reported the response options presented to participants, the response options were not those used in the 1985 version of the LSHS. Thus, for the sake of clarity and transparency, it would be useful for authors to report basic information about the scale they employed such as the response options presented to participants.

These findings are consistent with assessments of measurement practices in other sub-fields of psychological science where suboptimal measurement practices have been reported. This has been seen, for example, in depression (Mew et al., 2020), sexual behaviour (Kohut et al., 2020), and addiction (King et al., 2020) research, where a wide variety of different scales are used to assess the same variables-of-interest, and in social/personality science, where the scales employed in a study are often poorly described (e.g. in terms of the numbers of items and the response options presented to participants; Flake et al., 2017).

Our findings of suboptimal measurement practices and of variation in how HEs are measured have important implications for hallucinations research. Primarily, the combination of suboptimal measurement practices and the variation in how HEs are measured make it difficult to build a cumulative science, where others’ research can be reproduced and replicated, and where researchers can easily synthesise data collected across different studies. This may, for example, account for some of the inconsistent findings reported by hallucinations researchers, such as the association between the frequency of hallucinatory experiences and the vividness of mental imagery reported by non-clinical participants (e.g. Aynsworth et al., 2017; Mitrenga et al., 2019). We return to this issue in the General Discussion.

This study had several limitations. First, we limited our sample of reviewed papers to only 100. Clearly, we would be able to generalise our findings more broadly had we sampled a larger number of studies. That said, our aim was to examine current measurement practices in hallucinations research and including more studies in our review (e.g. 250) would have meant reviewing older publications, which would have meant that our analysis would not reflect the field’s current practices. Second, we excluded non-English versions of the LSHS from the review, as we were unable to verify whether meaningful changes had been made to the LSHS items as they were translated. It is unclear whether including non-English versions of the LSHS in the review would have increased or reduced the amount of variation in measurement practices we observed. Finally, our focus on the LSHS meant that we primarily reviewed studies involving non-clinical samples. It would be valuable to investigate measurement practices in hallucinations research with predominately clinical participants by, for example, examining variation in how measures such as the Psychotic Symptoms Rating Scale (Haddock et al., 1999) are employed.

## Study 2 introduction

Cognitive models (e.g. Bentall & Fernyhough, 2008) suggest that people who experience auditory hallucinations have a bias where they mistake internal, self-generated cognitions for external, non-self-generated events. Two main families of tasks have been employed to test this claim – source monitoring paradigms and signal detection paradigms. Across a series of trials, these tasks require participants to judge whether an item was internal and/or self-generated, or was external and/or non-self-generated. In source monitoring tasks, these judgements are made during a testing phase, where participants must remember events from an earlier encoding phase. In signal detection tasks, participants make these judgements “in real time”.

While meta-analytic studies (Brookwell et al., 2013) have reported that there are medium-to-large associations between performance on these tasks and the presence of hallucinations/HEs, findings from individual studies have been inconsistent. The use of small sample sizes presumably plays a role in the inconsistent effects reported across studies. For example, the median sample size in the clinical studies synthesised in Brookwell et al.’s meta-analysis was 30; this sample size would only give us reasonably precise estimates of effect sizes if the “true” association between task performance and presence/frequency of hallucinations/HEs was around ρ = .70 (Schönbrodt & Perugini, 2013). This issue may be compounded by suboptimal levels of reliability of the measures obtained from source monitoring and signal detection tasks. However, at present, it is unclear if this is the case, as we know very little about the reliability of the measurements obtained from source monitoring and signal detection tasks. The aim of Study 2, therefore, was to examine the reliability of the measurements obtained from source monitoring and signal detection tasks, using data from previously published studies. Given that we lack data that would allow us to examine test-rest reliability of the measures obtained from these tasks, we estimated the internal reliability of these measurements by examining their internal consistency.

## Study 2 method

### Datasets

We re-analysed data from four publications (five studies) – Smailes et al. (2015), Garrison et al. (2017), Alderson-Day et al. (2019), and Moseley et al. (2021). We selected these studies because we were able to access trial-level data from the tasks. Trial-level data is required to examine the internal consistency of the measurements obtained from a task (see Analyses section, below), and because these studies were authored/co-authored by at least one of the current study’s authors (BA-D, PM, or DS), we had full access to the datasets. In addition, we selected these studies because they are available as peer-reviewed publications, which allows us to explain their methods briefly here, and to direct readers to the original publications for more detailed information.

Two studies (Moseley et al., 2021; Smailes et al., 2015) employed signal detection tasks, and four employed source monitoring tasks (Garrison et al., 2017, Study 1; Garrison et al., 2017 Study 2; Alderson-Day et al., 2019; Moseley et al., 2021). In the Supplementary Materials we describe the tasks employed in brief (e.g. the number of trials employed), with more detail (e.g. the duration of each trial) available in the original publications.

Across all studies, we followed the approach recommended in Parsons et al. (2019) of reporting reliability estimates for variables that corresponded as closely as possible with the outcomes reported in previous research (e.g. we reported separate reliability estimates for the different groups created in Garrison et al., 2017, Study 1). For each study (or group within a study), we have reported two reliability estimates. For the signal detection tasks, we obtained a reliability estimate for the number of “hits” participants made, and a reliability estimate for the number of “false alarms” participants made. For the source monitoring tasks, we obtained a reliability estimate for the number of “internal misattributions” participants made (where a participant misremembered something that another person had generated or said, as something they had generated, said, or imagined), and a reliability estimate for the number of “external misattributions” participants made (where a participant misremembered something that they had generated, imagined, or said, as something another person had generated or said). In some instances, the number of participants reported in Table 4 differ slightly from the numbers reported in the original publication as the original publication may have only reported data from participants for which we had complete datasets for all of the variables measured in the study (in which case the N reported in Table 4 is larger than in the original publication), or because there were problems re-formatting some participants' data so that it could be used to calculate a reliability estimate study (in which case the N reported in Table 4 is smaller than in the original publication).

**Table 4.** Reliability estimates across studies, samples, tasks, and outcomes.

Study name	Task/Sample	N	*r_s_* (95% CI) for Hits	*r_s_* (95% CI) for False Alarms
Smailes et al. (2015)	SDT/Non-clinical	139	0.809 (0.783 to 0.836)	0.853 (0.823 to 0.883)
Moseley et al. (2021)	SDT/Non-clinical	594	0.808 (0.797 to 0.819)	0.939 (0.919 to 0.958)
Study Name	Task/Sample	N	*r_s_* (95% CI) for Internal Misattributions	*r_s_* (95% CI) for External Misattributions
Garrison et al. (2017) Study 1	SMT: Imagined vs Perceived/Non-clinical LP Group	22	0.632 (0.578 to 0.686)	0.393 (0.339 to 0.448)
Garrison et al. (2017) Study 1	SMT: Imagined vs Perceived/Non-clinical HP Group	25	0.598 (0.552 to 0.644)	0.781 (0.730 to 0.831)
Garrison et al. (2017) Study 1	SMT: Self- vs Other-Read/Non-clinical LP Group	22	0.569 (0.515 to 0.622)	0.676 (0.615 to 0.737)
Garrison et al. (2017) Study 1	SMT: Self- vs Other-Read/Non-clinical HP Group	25	0.526 (0.476 to 0.575)	0.434 (0.386 to 0.482)
Garrison et al. (2017) Study 2	SMT: Imagine vs. Say/Non-clinical	120	0.374 (0.352 to 0.396)	0.707 (0.684 to 0.729)
Alderson-Day et al. (2019)	SMT: Say vs. Hear/Non-clinical	76	0.588 (0.555 to 0.620)	0.667 (0.647 to 0.688)
Moseley et al. (2021)	SMT: Imagine vs. Hear/Non-clinical	594	0.695 (0.684 to 0.706)	0.679 (0.666 to 0.691)

Notes: SDT = Signal detection task; SMT = Source monitoring task; HP = Hallucination-prone; LP = Low-hallucination-prone; rs = Spearman-Brown corrected estimate.

### Analyses

Data (available at doi: 10.17605/osf.io/d3gnk) were analysed in R (R Core Team, 2018) using Sherman’s (2015) *multicon* package. This package estimates permutation-based split-half reliabilities for the measures obtained from tasks. The permutation-based split-half reliability is an estimate of a measure’s internal reliability, similar to Cronbach’s alpha. While Cronbach’s alpha can be calculated easily for measures obtained by questionnaires, it cannot often be calculated for tasks, because the order of trials is typically randomised and so varies across participants, and alpha can only be calculated when items are presented in a fixed order (Parsons et al., 2019). Split-half reliability refers to an estimate of reliability where the data from participants are divided in two (e.g. data from odd trials and data from even trials), and the correlation between these two halves is the calculated. This correlation is then used as an estimate of the measure’s internal reliability. While these estimates tend to be unstable, this can be addressed by repeatedly, randomly dividing the data in two, calculating the correlation between the two halves, and then finding the average of these correlations. The Spearman-Brown correction is then applied to account for underestimation of reliability that may result from halving the number of trials (Parsons et al., 2019). The permutation-based split-half reliability is this corrected average correlation. Here, 5,000 random splits were performed to obtain each reliability estimate.

## Study 2 results and discussion

As shown in Table 4, a range of reliability estimates were generated for the datasets we analysed. The reliability estimates for measures obtained using signal detection tasks were higher than the estimates for measures obtained using source monitoring tasks, although the confidence intervals around these estimates overlapped in some instances. Parsons et al. (2019) argue that reliability estimates are best treated as continuous variables but note that some categorical conventions of what should be classed as moderate, good, and excellent reliability do exist. According to the thresholds proposed by Koo and Li (2016), 10 of the 18 reliability estimates reported in Table 1 would be classed as moderate, four would be classed as good, and one would be classed as excellent. That said, others (e.g. Barch et al., 2007) have proposed that reliability estimates around .90 are optimal for cognitive tasks, and only one of the reliability estimates we report reached that threshold.

Our findings that the measurements obtained by some of the tasks employed in hallucinations research have suboptimal levels of reliability are consistent with data from other sub-fields of psychological science. For example, analyses of the reliability of measurements obtained by tasks employed in anxiety research (e.g. Kappenman et al., 2014) and in inhibition research (e.g. Hedge et al., 2018) suggest that they may have unacceptably low levels of reliability. That being said, our analyses suggest that the measures obtained using signal detection tasks may be more reliable than those obtained using source monitoring tasks and this is consistent with other data that has suggested that measures obtained using paradigms similar to signal detection tasks have good levels of test-retest reliability (e.g. Huque et al., 2017 ).

These findings have important implications for hallucinations research. In contexts where researchers are investigating “true effects”, measuring variables with sub-optimal levels of reliability results in the attenuation of “true associations” and so statistical power is reduced (Parsons et al., 2019; Rouder et al., 2019). This is especially unfortunate in hallucinations research where researchers may find it difficult to recruit large samples. In contexts where researchers are not investigating “true effects” the reduction in statistical power caused by using measurements with suboptimal levels of reliability increases the likelihood of false-positive findings (Bakker et al., 2012). Thus, in two ways, it is possible that this issue of sub-optimal levels of reliability has contributed to some findings in our field being difficult to replicate (e.g. inconsistent associations between atypical source monitoring and HEs; Brookwell et al., 2013; Moseley et al., 2021).

This study had several limitations. First, the set of studies we re-analysed data from did not include a clinical sample. The measurements obtained by some neuropsychological tasks are more reliable in clinical than in non-clinical samples (Kopp et al., 2021), and it is possible that this may also be true for measures obtained using source monitoring and signal detection tasks. Future research should examine if this is the case. Second, our analyses focussed on external and internal misattributions (for the source monitoring tasks) and on hits and false alarms (for the signal detection tasks). These are the outcomes we often employ and are the outcomes for which we could calculate split-half reliability estimates for. However, many researchers use signal detection parameters as indices of performance on signal detection and source monitoring tasks, and we cannot comment on the reliability of those measurements. This is because signal detection parameters (e.g. d-prime, beta) are task-level summary scores, whereas split-half reliability estimation relies on trial-level data. That said, d-prime and beta are calculated using hit-rate and false alarm-rate, and so the reliability of those variables should be of interest to researchers who analyse source monitoring and/or signal detection task performance in terms of signal detection parameters. Nevertheless, it would be valuable for future studies that employ source monitoring and/or signal detection tasks to establish the test-retest reliabilities of signal detection parameters.

## General discussion

Across two studies, we have provided evidence that hallucinations researchers (including some of the authors of this article) engage in suboptimal measurement practices by, for example, using a wide range of different questionnaires to assess the same variable across different studies, modifying validated questionnaires, failing to adequately describe the measures that have been employed, and by using tasks that measure variables-of-interest with low levels of reliability. The primary consequences of these sub-optimal measurement practices are likely to be a reduction in the reproducibility and replicability of the findings hallucinations researchers report. For example, when it is unclear what version of the LSHS has been used in a study, it is harder for that study’s methods to be reproduced by other researchers aiming to replicate the initial study’s findings. Meanwhile, when measurements with low reliability are obtained, statistical power is reduced, and this increases the likelihood of a study reporting false-negative and false-positive findings. Together, these issues reduce the robustness and credibility of the findings generated by hallucinations researchers.

An implication of these findings is that hallucinations researchers need to attend to issues of measurement more carefully. It should, perhaps, become the norm to report the reliability of the measurements obtained by tasks employed in hallucinations research. Several R packages now exist that allow researchers to estimate the internal reliability of measures obtained from tasks (e.g. splithalf and multicon; Parsons, 2019; Sherman, 2015), and the Excel-based tool RELex (Steinke & Kopp, 2020) can be used by researchers who are unfamiliar with using R. The availability of these packages/tools should facilitate the reporting of reliability estimates. In addition, the scales employed in a study should be described more comprehensively and accurately. Where word-limits prevent researchers from describing the scales employed in detail, the Open Science Framework (osf.io) can be used to post study materials, so that scales which must be reported briefly in an article can be described/presented in full elsewhere. Finally, it seems undesirable to encourage all hallucinations researchers to employ the same measures of hallucinatory experiences (e.g. see Patalay & Fried, 2021, on possible unintended consequences of mandating the use of specific measures for assessing depression and anxiety). Instead, following the recommendation proposed by Flake and Fried (2020), researchers should be encouraged to be more explicit and precise when discussing and describing their construct(s)-of-interest. In terms of hallucinations research, this may involve, for example, avoiding the use of terms such as “hallucination-proneness” in future, as this term may have different meanings for different researchers (e.g. the frequency of experiencing a narrow set of hallucinatory percepts, such as hearing sounds that others do not, versus the frequency of experiencing a broader set of percepts/cognitions that may be related to hallucinations, such as intrusive thoughts or vivid daydreams). Instead, researchers should be encouraged to define their construct-of-interest more precisely and should explain how the scale they have selected measures that construct effectively. Again, where word-limits prevent this kind of reporting in the Method section of a journal article, the Open Science Framework can be used to publish supplementary methodological information.

More broadly, while engaging in better measurement practices would improve the rigour of hallucinations research, it should be considered as only part of a wider set of reforms that the field should engage in. Clinical psychological science has been slow to adopt the reforms that other sub-fields of psychology have engaged in (Tackett et al., 2019), such as the use of pre-registration, open data-sharing, and the use of open-source materials. Given the importance of generating a trustworthy evidence base from which interventions that help people with distressing hallucinations can be developed, it would be extremely valuable if hallucinations researchers engaged in this wider set of methodological reforms, as well as engaging in better measurement practices.

## Supplementary Material

Supplemental Material
