# Supplementary material for: Measurement practices in hallucinations research
Source: Cogn Neuropsychiatry. 2021 Nov 8;27(2-3):183–98. doi: 10.1080/13546805.2021.1999224 (PMC9006980; doi:10.1080/13546805.2021.1999224)
Supplement: Supplemental Material [file PCNP_A_1999224_SM5024.docx]

**Measurement Practices in Hallucinations Research Supplementary Materials**

**Further Information on Datasets Analysed in Study 2**

We re-analysed data from four publications (five studies) for which we were able to access trial-level data. In the main article, we described the studies very briefly. Here, we discuss each study in a little more detail. Readers are directed to other publications for full details about the study methods.

Two reliability estimates (corresponding to ‘hits’ and ‘false alarms’, see below) were obtained using data from Smailes et al. (2015). In this study, non-clinical participants completed a 60-trial auditory signal detection task, which required them to detect speech sounds in white noise. On 12 trials, the speech was easy-to-detect, on 22 trials, the speech was difficult-to-detect, and on 26 trials, no speech was presented. Participants, therefore had 34 opportunities to make a ‘hit’ (detecting speech when it was present) and 26 opportunities to make a false alarm (detecting speech when it was not presented).

Two reliability estimates were obtained using data from Moseley et al. (2020). In this study, participants completed a 72-trial auditory signal detection task, which was similar to the task employed in Smailes et al. (2015), but which differed in the number of different trial types (here there were 12 trials in which speech was easy-to-detect, 24 trials in which speech was difficult-to-detect, and 36 trials in which no speech was presented).

Eight reliability estimates were obtained using data from Garrison et al. (2017) Study 1. In this study, participants completed a source monitoring task, which involved a 144-trial encoding phase across six task blocks. During this phase, participants were presented with word-pairs, which were either complete (‘bacon and eggs’) or incomplete (‘salt and p_____’). Participants were instructed to either listen to the researcher read aloud the word-pair, or to read aloud the word-pair themselves. In the testing phase, participants were presented with all 144 word-pairs from the encoding phase, as well as 72 distractor word-pairs and had to judge whether (a) the word-pair was new, or had been read aloud by themselves or read aloud by the researcher and (b) whether the second half of the word-pair had been present (i.e., they had perceived it), or if it had been absent (i.e., they had imagined it). Participants, therefore, could make two types of internal misattributions (judging that they had read the item, when it had been read by the researcher; judging that they had imagined the item when it had been perceived) and two types of external misattributions (judging that the researcher read the item when the participant had read it aloud; judging that they had perceived the item when they had imagined it). There were 72 opportunities to make each type of misattribution. The eight reliability estimates refer to these four error types for both ‘high’ and ‘low’ hallucination-prone groups reported by Garrison et al. (2017).

Two reliability estimates were obtained using data from Garrison et al. (2017) Study 2. In this study, participants completed a source monitoring task, which involved a 72-trial encoding phase. During this phase, participants were presented with word-pairs, and were instructed to either to say the word-pair in inner speech or to say the word-pair ‘out loud’. In the testing phase, participants were presented with all 72 word-pairs from the encoding phase, and had to judge whether the word-pair had been said in inner speech, or had been said out loud. Participants, therefore, had 36 opportunities to make an internal misattribution (judging that they had said the item in inner speech, when they had said the item out loud) and 36 opportunities to make an external misattributions (judging that they had said the item out loud, when they had said the item in inner speech).

Two reliability estimates were obtained using data from Alderson-Day et al. (2019). In this study, participants completed a source monitoring task, which involved a 48-trial encoding phase. During this phase, participants were presented with word-pairs, and were instructed to either listen to the word-pair be read aloud over headphones, or to read aloud the word-pair themselves. In the testing phase, participants were presented with all 48 word-pairs from the encoding phase, as well as 24 distractor word-pairs and had to judge whether the word-pair was new, or had been read aloud by themselves, or if they had listened to it being read aloud. Participants, therefore, had 24 opportunities to make an internal misattribution (judging that they had read the item, when it had been read to them) and 24 opportunities to make an external misattributions (judging that the item had been read to them, when they had read the item).

Two reliability estimates were obtained using data from Moseley et al. (2020). In this study, participants completed a source monitoring task, which involved a 48-trial encoding phase. During this phase, participants were presented with words, and were instructed to either listen to the word be read aloud over headphones, or to imagine reading the word aloud. In the testing phase, participants were presented with all 48 words from the encoding phase, as well as 24 distractor words and had to judge whether the word was new, or if they had listened to it being read aloud, or if they had imagined reading the word aloud. Participants, therefore, had 24 opportunities to make an internal misattribution (judging that they had imagined the item being read, when it had been read to them) and 24 opportunities to make an external misattributions (judging that the item had been read to them, when they had imagined the item being read).

**References**

Alderson-Day, B., Smailes, D., Moffatt, J., Mitrenga, K., Moseley, P., & Fernyhough, C. (2019). Intentional inhibition but not source memory is related to hallucination-proneness and intrusive thoughts in a university sample. Cortex, 113, 267-278.

Garrison, J. R., Moseley, P., Alderson-Day, B., Smailes, D., Fernyhough, C., & Simons, J. S. (2017). Testing continuum models of psychosis: No reduction in source monitoring ability in healthy individuals prone to auditory hallucinations. *Cortex, 91,* 197-207.

Moseley, P., Aleman, A., Allen, P., Bell, V., Bless, J., Bortolon, C., ... & Fernyhough, C. (2020). Correlates of hallucinatory experiences in the general population: an international multi-site replication study. *Psychological Science.*

Smailes, D., Meins, E., & Fernyhough, C. (2015). Associations between intrusive thoughts, reality discrimination and hallucination-proneness in healthy young adults. *Cognitive Neuropsychiatry, 20,* 72-80.
